# The Knowledge Acquired by Parents in Pregnancy Monitoring Consultations

**DOI:** 10.3390/ijerph21080967

**Published:** 2024-07-24

**Authors:** Raquel Cerdeira, Otília Zangão

**Affiliations:** 1 Hospital CUF Descobertas, 1998-018 Lisboa, Portugal; raquelmcerdeira@gmail.com; 2 Unidade Local de Saúde de Almada-Seixal, 2805-267 Almada, Portugal; 3 Department of Nursing, Higher School of Nursing, University of Évora, 7000-811 Évora, Portugal; 4 CHRC—Comprehensive Health Research Centre, NOVA Medical School, University of Évora, 1150-082 Lisboa, Portugal

**Keywords:** fathers, pregnancy, prenatal care, prenatal education, health literacy

## Abstract

(1) Pregnancy surveillance nursing consultations in the pandemic context have undergone some changes, namely the restriction of accompanying persons. In this sense, assessing the knowledge acquired by parents in pregnancy surveillance is of great importance. Since there are no studies on the subject in the period before the pandemic, we will only assess the knowledge acquired by parents in pregnancy monitoring. (2) For this reason, the aim of our study is to evaluate the knowledge acquired by parents in pregnancy surveillance. (3) This study is descriptive, cross-sectional and quantitative in nature, with a sample of 196 couples. A questionnaire was administered about the knowledge acquired by parents in pregnancy surveillance nursing consultations. (4) Pregnant mothers and their partners reported having some knowledge. The classification was assessed using a questionnaire with quantifiable response options from 1 to 5 points. Items with a score of 1 indicate a low level of knowledge and 5 indicates a higher level of knowledge. (5) We can verify that the level of knowledge acquired by the pregnant mother during the pregnancy surveillance nursing consultation is higher than the level of knowledge acquired by the father during the same consultation. Most parents consider it important to receive information through the pregnancy surveillance nursing consultations carried out by Maternal and Obstetric Health Nurse Specialists (midwives).

## 1. Introduction

In recent years, the presence of fathers in pregnancy surveillance has emerged in debates, most importantly, a change of perspective has emerged on the part of researchers, managers and health professionals. The presence of the father during birth is clearly a cultural matter and is currently a common practice worldwide. These changes have contributed to an increase in the paternal bond with both the mother and the baby. Including the father in prenatal care is a strategy that provides greater interest in the pregnancy, encourages the father to take greater care of the mother and the baby, increases affective family emotional involvement and increases the level of knowledge, helping with a positive transition to parenthood [1].

Fathers can provide important psychological and emotional support for mothers during pregnancy and childbirth; in particular, they can reduce pain, panic and exhaustion during labor. The World Health Organization (WHO) also shows that involving men in maternal and child health programs can reduce maternal and child mortality during pregnancy and childbirth by being prepared, for example, for obstetric emergencies. Increasing involvement in fatherhood can also benefit fathers’ own health and well-being. For example, fathers who have recognized their new position as fathers and had emotional support during pregnancy have better mental and physical health [1].

In this sense, Nurse Specialists in Maternal and Obstetric Health Nursing (midwives) have an important role in health education, not only in encouraging the father to attend prenatal care appointments, but also in the transmission of specialized information in the area of maternal health as a reliable, updated and scientific source. The midwife has an important role in integrating the couple into this new phase of their lives, making the parenthood process easier. The midwife should base prenatal education on the individual needs and concerns of each couple, as well as providing emotional support in the absence of parents at prenatal surveillance appointments [1].

Knowledge is defined as the specific content of thought, based on wisdom, information and learned and acquired skills [2]. Knowledge includes the understanding of certain information and the ability to mobilize information. Knowledge is understood as a facilitating condition in transition processes, such as the transition to parenthood [3]. There are many definitions that emerge in the area of knowledge and that are related to each other, namely health literacy and empowerment.

Health literacy (HL) is defined by the WHO as personal knowledge and personal skills that accumulate through daily activities, social interactions and generations. Personal knowledge and skills are mediated by organizational structures and the availability of resources that enable people to access, understand, analyze and use information and services to promote and maintain good health and well-being for themselves and others [4]. HL is also understood as “the ability to make informed decisions about health, in everyday life, and also with regard to the development of the Health System, as it contains essential elements of the educational process and provides indispensable skills for self-care” [5] (p. 27). HL is the key to empowering people in the health field [6].

Limited health literacy is related to higher healthcare costs and higher hospitalization rates [7]. It is understood today that HL becomes a fundamental principle for health promotion. It can make a difference, particularly for people with minority cultural and linguistic backgrounds or with a socioeconomic status that makes access to health services difficult. However, putting this principle into practice presents significant challenges to healthcare professionals due to the resource constraints they face in providing care, including inadequate knowledge about the importance of literacy [8].

Maternal health literacy (MHL) refers to the knowledge and ability of pregnant mothers to detect risk factors, identify warning signs during pregnancy that could become complications and adopt a healthy lifestyle that includes adequate nutrition [9,10,11]. It is therefore important that throughout pregnancy, the pregnant mother acquires the knowledge and autonomy to make the right choices regarding their own health.

According to the WHO [12], MHL among mothers is increasing, which means that they are learning more about birth, which empowers them. Educational tools such as childbirth preparation groups and information leaflets are promoted, which helps them in making informed decisions. Supporting a mother’s right to make decisions about prenatal care is very important, but it is imperative that health professionals publicize the risks and benefits and explain the options. These aspects should be discussed continually with the mother during the prenatal period, enabling her to assimilate all of the information and be able to make an informed decision.

According to the Order of Nurses (the ON is an organization from Portugal), the specialist nurse is provided with in-depth knowledge in the specific field of nursing, both in the promotion and treatment of health. The specialist nurse demonstrates high levels of knowledge and specialized skills relating to a field of intervention. Therefore, midwives integrate a set of specialized clinical skills in the area of Maternal and Obstetric Health Nursing, thus being the health professional with the main role in transmitting knowledge, empowerment and health education [5].

Therefore, the research question formulated for this study was “What knowledge have parents acquired about pregnancy surveillance?”.

## 2. Materials and Methods

This is a descriptive cross-sectional study of a quantitative nature, with a sample of 196 couples. A questionnaire was administered about the knowledge acquired by parents during nursing consultations.

The type of sample was non-probabilistic and of convenience, based on pregnant couples monitored in an external obstetrics consultation at a private hospital in Lisbon during cardiotocographies (CTGs).

The inclusion criteria were as follows: fluency in Portuguese, age over 18 years, pregnant couples monitored in an external consultation at a Lisbon private hospital, participating voluntarily and having access to the internet to complete the questionnaire. The exclusion criteria were as follows: couples of foreign nationality with no fluency in Portuguese, reference to mental/psychiatric illness and pregnant mothers under the age of 18 years.

To calculate the sample size, we used the Creative Research Systems (https://www.surveysystem.com/sscalc.htm (accessed on 5 January 2021)). In 2019, 5124 CTGs were carried out, and on average, in a month, 427 CTGs were performed. With a confidence level of 95% and a confidence interval of 7, the sample size would be 189 participants. This means that with this confidence level and the confidence interval together, you can say that you are 95% sure that the true percentage of the population is between 43% and 51%.

The sample consisted of 196 couples who met the eligibility criteria and attended a consultation between February and June 2021. All of the ethical principles inherent in this type of investigation were assured and guaranteed. Authorization was requested from the institution’s Board of Directors and Ethics Committee, and a favorable opinion was obtained (project/study 87).

Data collection was divided into two sections. The first referred to sociodemographic variables (age, marital status, religion, nationality, educational qualifications) and obstetric variables (number of births, planned pregnancy, desired pregnancy, location of pregnancy monitoring, performance of childbirth preparation course (CPC), if the couple had a companion during pregnancy monitoring appointments). The second section consisted of the questionnaire about the knowledge acquired by the couple during pregnancy monitoring nursing appointments. This questionnaire was constructed as part of this study and was based on the prenatal education carried out by the midwives during nursing consultations throughout the three trimesters of pregnancy at a Lisbon private hospital. Each item describes the prenatal education carried out in each trimester. The classifications were rated according to a 5-point Likert scale of “I have no knowledge”, “I have little knowledge”, “I don’t know/No answer”, “I have knowledge” and “I have a lot of knowledge”. The items were scored from 1 to 5, with higher scores indicating a higher level of knowledge.

To check the level of knowledge acquired by the couple during pregnancy follow-up appointments, a questionnaire was used during the appointments based on the Pregnancy Health Bulletin (PHB) of a private hospital of Lisbon. During the course of this study, research was carried out to find an instrument that would meet the requirements. No validated questionnaire was found that responded to the phenomenon under study in the population and context of the research; so, this questionnaire, based on the questions in the PHB, made it possible to respond to the object of the present study. In this case, the creation of this questionnaire was necessary to address specific issues that were not covered by existing instruments.

Since this questionnaire was built based on the PHB used in the services for pregnant mother, it was decided to build the questionnaire for fathers with the same items. Therefore, since this questionnaire was constructed using an instrument already in use at the institution and for a population with these characteristics, it seems to us that it complies with the recommendations.

The self-administered questionnaire was filled in by the couples themselves via the internet and was made available by a link sent by email and/or a QR code provided in sticker format, pasted on the pregnant mother’s report card. The data were collected using the Google Forms platform. We measured parents’ knowledge when they started CTGs at around 36 weeks of pregnancy. Couples were approached to answer the questionnaire based on the content discussed at antenatal care appointments. The topics covered in the educational content during pregnancy were as follows: the importance of vaccination; 1st trimester discomfort (nausea, vomiting, constipation, drowsiness); the need for a healthy, fractioned diet; sexuality; the need for rest periods throughout the day; the dangers of self-medication; body hygiene; clothing and footwear; maternity layette; choosing a place of birth/scheduling a visit to the maternity hospital; birth preparation courses; advantages of breastfeeding; preparing the nipple for breastfeeding; correct posture during feeds; technique, duration and intervals between feeds; 2nd trimester discomfort; signs and symptoms of urinary incontinence; the importance of birth preparation and signs of labor; antenatal consultations with the pediatrician; explanations of the CTG and its importance; teaching how to fill in the fetal movement chart; preparing a suitcase for the hospital; infant deafness test; the importance of monitoring the newborn; the baby’s foot prick test; vaccinating the baby; what is puerperium; lochia, puerperal depression, uterine involution, rising milk and postpartum consultations.

The data were processed using the IBM SPSS Statistics program, version 24. The data were processed and analyzed using descriptive and inferential statistics. Absolute and relative frequencies were used; measures of central tendency (mean, median and mode), measures of dispersion or variability (standard deviations, minimum value and maximum value), the parametric test (Student’s t), Pearson’s correlation coefficient or Pearson’s r and Cronbach’s alpha coefficient were used for this study.

## 3. Results

This study consisted of a sample of 196 couples, and the results are presented below according to sociodemographic characteristics, obstetric and gynecological characteristics, and the mother’s knowledge and fathers’ knowledge.

- Sociodemographic characteristics of the study participants

It was found that the highest prevalence in the sample was couples who were married (88.3%), with an average age between 31 and 35 years (39.6%) in the case of the pregnant mother and between 36 and 40 years in the case of the father (33.5%), of Portuguese nationality (98%), of Caucasian race, of Catholic religion (80.7% in the case of the pregnant mother and 70.6% in the case of the father), and with a university degree (47.2% in the case of the pregnant mother and 41.6% in the case of the parents) (Table 1).

- Obstetric and gynecologic characteristics

With regard to the obstetric profile, it was found that the majority of the mothers were primiparous (45.2%), the pregnancy was planned (81.7%), desired (98.5%) and monitored (100%) in a private hospital (70.1%), and most couples had not taken any childbirth preparation courses (52.8%). The reasons given were that “they had done it in a previous pregnancy”, “they didn’t think it was necessary as a couple”, “due to COVID-19”, “it wasn’t possible to do it in person” and “they couldn’t get a place in time”. In relation to the current pregnancy, the majority of mothers were not accompanied by their significant other (97.5%) and all of the parents who reported not being present at appointments and ultrasounds gave the COVID-19 pandemic as the reason. The average gestational age of the mothers who answered the questionnaire was 36 weeks and the average number of pregnancy monitoring appointments was nine (Table 2).

- Mother’s knowledge

The majority of mothers showed that they had a good level of knowledge, responding mostly to level “4—I have knowledge”. No knowledge was considered insufficient (below level 3—“I don’t know, I don’t answer”). The strongest knowledge was “Discomforts in the 1st Trimester (nausea, vomiting, constipation, drowsiness)” (4.36), “Need for a healthy and fractioned diet” (4.31) and “The importance of vaccination” (4.18) (Table 3).

- Father’s knowledge

The knowledge acquired by the father considered insufficient (below level 3—“I don’t know, I don’t answer”) concerned “Preparing the nipple for breastfeeding”, “Technique, duration and intervals between feedings”, “Signs and symptoms of urinary incontinence”, “Teaching about filling out the fetal movement chart”, “Postpartum, what is it?” and “Lochia, puerperal depression, uterine involution, milk letdown, postpartum consultation”. The strongest areas of knowledge were “Advantages of breastfeeding” (3.88), “Need for healthy and fractioned diet” (3.85), “The importance of vaccination” (3.78) and “Dangers of self-medication” (3.75) (Table 4).

The majority of parents obtained this knowledge through the pregnant mother (82.7%) and the internet (36.5%), followed by “Books, Magazines and articles” (25.9%), “Pre-delivery preparation course” (22.6%), “Previous experience” (7.1%), “At the Nursing consultation” (6.1%), “Friends/family” (1.5%) and “Profession” (1.5%), while some stated that “I did not acquire knowledge (1%) (Table 3).

In relation to the most positive aspects of the nursing consultation, the majority suggested “Clearing doubts/obtaining information”, “Everything positive” and “Attention, friendliness, proximity and interest from nurses”. The most frequently mentioned responses regarding the most negative aspects of nursing consultations were “No”, “Consultations too quick” and “Lack of information”. Most parents consider it important to receive information through nursing consultations, they felt sad due to their absence from consultations/examinations during pregnancy, they would have felt more involved if they had been present at the consultations and they would have experienced the pregnancy with more joy. Both the mother’s knowledge and father’s knowledge correlate positively and statistically significantly with all dimensions. The majority of couples reported having knowledge (62.4%). Regarding the level of knowledge, couples reported having some knowledge, although it was not very high.

The limitations of the present study are the small sample size and the sampling method, which was non-probabilistic and adapted to the time, available resources and type of study design, which could limit the generalization of the data. Another limitation that could be considered is the fact that when the questionnaire is filled out on-line, parents can bias their answers and in turn the results. Another limitation is that we did not use a validated scale on knowledge, since it did not exist.

## 4. Discussion

### 4.1. Pregnant Mothers’ Knowledge

In this study, on average, pregnant mothers reported having some knowledge, with a score of 3.84, although it was not very high (1—I have no knowledge and 5—I have a lot of knowledge). This is in line with studies which state that pregnant mothers who are accompanied by their partners receive effective support; their presence is useful because it supports and promotes the feeling of paternity and helps with health education and communication between the couple [13,14]. Paternal participation in consultations increases maternal and paternal knowledge. Their presence provides a more humanized moment during pregnancy and prepares the couple for the transition to parenthood. The father’s presence makes the pregnant mother safer and increases the closeness between the couple; the skills and knowledge they acquire during consultations will help them to help the pregnant mother, undertaking an active role in the pregnancy [15]. When the father is present, he will remember the information that was provided and help the pregnant mother to retain the information; therefore, this is reflected in the knowledge acquired by both.

The majority of mothers showed that they had a good level of knowledge, responding mostly to level “4—I have knowledge”. No knowledge was considered insufficient (below level 3—“I don’t know, I don’t answer”). The strongest areas of knowledge were as follows:

- “Discomforts in the 1st Trimester (nausea, vomiting, constipation, drowsiness)” (4.36);
- “Need for a healthy and fractioned diet” (4.31);
- “The importance of vaccination” (4.18).

A study on the knowledge and acceptance of vaccination against influenza and whooping cough among pregnant mothers in Turkey reveals that there is an important role played by health professionals in disseminating information/knowledge about whooping cough and influenza vaccination to improve the acceptance of vaccination during pregnancy [16]. Another study evaluating pregnant mothers’ knowledge about pregnancy care in Brazil demonstrated that all pregnant mothers were informed that it was important to have vaccinations during pregnancy [17]. This study corroborates the results of our study, as both pregnant mothers and the parents collectively showed knowledge about the importance of vaccination. In the same study, 96% of pregnant mothers thought it was important to eat a healthy diet during pregnancy, while 4% thought it was irrelevant. All of the pregnant mothers thought that physical activity, such as walking, would not harm them or their babies; however, 58% of them believed that there were no dietary restrictions while they were pregnant [17]. In our study, both pregnant mothers and the fathers demonstrated knowledge of a balanced and healthy diet.

Furthermore, we found in our study that during pregnancy, the majority of mothers did not take any birth preparation courses (52.8%), and the majority of fathers did not either (63.5%). The reasons given were that “they had taken it in a previous pregnancy”, “they didn’t think it was necessary as a couple”, “due to COVID-19”, “it wasn’t possible to attend”, “it wasn’t in person” and “they couldn’t get a place in time”. Another study on pregnant and postpartum mothers’ knowledge of primary healthcare in Brazil showed that 80% participated in educational activities during prenatal care; however, only 46% said that they received some educational information during pregnancy. Of the information received, 8% received guidance on diet, 10% on diet and exercise, 20% on breastfeeding, 4% on family planning, 2% on prenatal care and 2% on vaccination; 98% know that smoking and/or drinking alcohol is harmful during pregnancy; 96% know to take vitamins such as ferrous sulphate and folic acid during pregnancy; 62% did not take any medication during pregnancy [18]. Other works recommended that childbirth preparation courses allow the pregnant mother to acquire basic knowledge, which helps them to develop independence, creating a sense of security for the moment of childbirth, giving them an active role and eliminating the passivity often assumed by pregnant mothers due to feelings of fear and insecurity [19].

### 4.2. Fathers’ Knowledge

The areas of knowledge acquired by the father considered insufficient (below level 3—“I don’t know, I don’t answer”) were “Preparing the nipple for breastfeeding”, “Technique, duration and intervals between feedings”, “Signs and symptoms of urinary incontinence”, “Teaching about filling out the fetal movement chart”, “Puerperium, what is it?” and “Lochia, puerperal depression, uterine involution, milk supply, postpartum consultation”. The strongest areas of knowledge were “Advantages of breastfeeding” (3.88), “Need for healthy and fractioned diet” (3.85), “The importance of vaccination” (3.78) and “Dangers of self-medication” (3.75).

A study on self-medication and the knowledge of pregnant mothers attending primary healthcare centers in Indonesia found that the occurrence of self-medication during pregnancy was low (11.7%). This study indicates relatively high levels of knowledge of the risk of using medicines during pregnancy, meaning mothers are less likely to self-medicate. These results are similar to those of other studies carried out in the Netherlands (12.5%), Nigeria (22.3%) and Saudi Arabia (13.2%) [20]. According to the results of another study carried out in Italy, it was observed that pregnant mothers are more likely to consult a health professional before taking over-the-counter medicines. This behavior is healthy and allows health professionals to pass on correct information about the use of medicines during pregnancy. The transmission of knowledge through health professionals about the potential risks of using non-prescribed medicines during pregnancy can help pregnant mothers to manage their use of over-the-counter medicines more safely [20]. This study corroborates our results: the men showed that they were aware of the dangers of self-medication, despite not having attended pregnancy monitoring appointments. We know that the majority of the knowledge they acquired during the pregnancy was obtained from the information passed on by the pregnant mother (82.7%) and from the internet (36.5%). We can assume that they may have acquired this knowledge from the pregnant mother. This result is in line with the study by Smyth [15], which states where and how men prefer to receive information; the literature, the internet and female partners were named as primary sources of information, while other men in a study commented that information obtained from these sources was as valuable as information obtained in prenatal education classes. However, there is no study aimed at assessing the knowledge acquired by fathers during pregnancy monitoring consultations.

The majority of couples reported having knowledge (62.4%) and the most mentioned topic they would like to be addressed was breastfeeding (29.9%). We can verify that the level of knowledge acquired by the pregnant mother in the prenatal surveillance nursing consultation is higher than the level of knowledge acquired by the father during pregnancy surveillance consultations. We can assume that this phenomenon is due to the fact that the father was unable to be present at nursing appointments due to the COVID-19 pandemic. According to Ferreira [21], the absence of the father is a risk factor for pregnancy, as evidence shows that pregnancy progresses better when the father participates in the pregnancy. Parents must participate in various ways during pregnancy, directly through their presence during ultrasounds and consultations, through praise and talk between couples, and when participating in domestic tasks, especially if they have other children [22], or indirectly by providing fundamental support for the pregnant mother and expressing their emotional support. Small gestures, such as conveying security to the mother and collaborating with the organization accommodating the baby’s arrival, can establish solid bonds between the couple/family and ensure that future generations are emotionally balanced, safe and happy [22].

The father has the right to accompany the pregnant mother and to be welcomed by health professionals. In Portugal, the father has the right to three days off work to accompany the pregnant mother to prenatal consultations [23]; however, we know that three days is insufficient to ensure that the benefits of the paternal presence during prenatal surveillance consultations are effective. The absence of the father can occur for several reasons: for work reasons, due to appointment times being at the same time as working hours; for conflicting reasons between couples, due to an unwanted and/or unplanned pregnancy; for family management reasons, having other children to care for and accompany; due to feelings of exclusion in consultations, as the father feels that the focus in consultations is on the mother and ends up giving up on accompanying them, among others [15]. However, in this study, it was found that all parents justified their absence from appointments for reasons due to the COVID-19 pandemic. In this period in which several physical, psychological and social changes arise in the life of the mother/couple, it is extremely important that both family members and healthcare professionals take care of this new family and establish a bond of trust and security [14]. The presence of the father must be encouraged by midwives. Information must be made available during consultations to prepare the father for the pregnancy, empowering and guiding him on his rights and allowing him to better understand the changes that will emerge in this new phase [15]. Their absence from consultations means that the information received by the pregnant mother is not transmitted in the same way at the same time with the same interlocutor to the father, which can lead to a loss of information. The pregnant mother may pay more attention to certain information that is more interesting to her and there is the possibility of losing information that could be seen as important to her partner. Since the level of knowledge acquired by the father is not null, this demonstrates that some knowledge is previously acquired, perhaps through sharing information from friends and family and/or even through their own experience if it is not the first pregnancy they have been involved with. However, there is an insufficient level of knowledge among fathers, as health education is aimed more at the mother. All of these topics are covered in the nursing consultations, which are relevant and appropriate for each quarter (1st T, 2nd T and 3rd T consultation), carried out by a midwife.

The father’s absence from prenatal surveillance consultations can influence the decrease in the level of knowledge acquired by fathers throughout the pregnancy. We know that the presence of the father during pregnancy surveillance consultations is beneficial [15].

### 4.3. Principal Findings

This article aimed to evaluate the knowledge acquired by parents in pregnancy surveillance consultations. On average, mothers (3.84) acquired more knowledge than fathers (3.36). It cannot be said that these results are unexpected, since the COVID-19 pandemic has changed healthcare. Since the parents were not present during the pregnancy surveillance appointments, these are subjects that parents can learn about through health education with midwives. However, the average value of knowledge acquired is not very high.

Inadequate health literacy (when compared to adequate health literacy) is strongly related to a low level knowledge or understanding of both care services and health outcomes. It may also be associated with a high probability of hospitalization [24,25]. A low level of health literacy has been identified in several studies as a risk factor for various diseases [26]. Adequate levels of health literacy seem to result in improvements in people’s health status [27].

According to a European health literacy survey carried out in 2014, 61% of the Portuguese population has a problematic or inadequate level of general health literacy. This means that more than half of the Portuguese population has difficulties in accessing, understanding and using health resources [28].

During pregnancy, the majority of mothers did not take any childbirth preparation courses (52.8%), and the majority of fathers did not either (63.5%). The reasons described were that “they had it done in a previous pregnancy”, “they didn’t think it was necessary as a couple”, “due to COVID-19”, “time was not possible”, “it wasn’t in person” and “they didn’t get a place in time”. It is understandable that as it is not the couple’s first pregnancy, they do not want to repeat the childbirth preparation courses, thinking that they have already acquired the necessary knowledge. However, each pregnancy is unique and develops differently. Childbirth preparation courses are fundamental for adjusting expectations, reformulating meanings, improving knowledge and developing skills, allowing each mother/couple to become the protagonist of their own childbirth [29].

In this study, the majority of mothers are graduates (47.2%) and the majority of fathers are too (41.6%). However, the fact that the level of education is high does not always mean that the level of health literacy is also high. The pregnancy period should be seen as an important opportunity to improve the health literacy of mothers, especially those who are socioeconomically disadvantaged [30]. The applicability of cheap and effective precautions that may affect children’s health and survival is associated with the educational level of mothers [31]. In the literature, there are studies examining the relationship between health literacy and health outcomes, whereas there is no study aiming to determine the effects of health literacy on a mother’s reproductive health.

Maternal health literacy needs to be emphasized because it affects a mother’s reproductive health. Information on preventive care, contraception, safe sex practices, and healthy pregnancy and postpartum practices is important for mothers to maintain their healthy and productive lives [31]. Pregnancy is an excellent time to increase maternal health literacy. At this stage, mothers use health services more frequently and are open to learning health-related knowledge and behaviors.

The results of the present study and also other studies in the literature clearly suggest that health literacy has a direct effect on pregnancy in terms of both the mother and infant and it is important to ascertain and improve the health literacy level of mothers.

## 5. Conclusions

Due to epidemiological and pandemic issues, restrictions were placed on the father’s presence in prenatal surveillance consultations in hospital institutions. Therefore, it is important to understand the level of knowledge that was acquired by the parents, since the father was absent from the nursing consultations for monitoring the pregnancy, and where this was acquired. A lack of knowledge and the father’s absence during prenatal surveillance consultations can influence the pregnancy. Pregnancy monitoring nursing consultations have a very strong significance in terms of health education, in the transmission of appropriate knowledge, in helping with the transition into the new parental role and gaining autonomy, and in restructuring and adapting to parenthood.

It would be a good suggestion to carry out more studies on this topic so we are able to compare and obtain the results of the level of knowledge acquired by the couple before and after the COVID-19 pandemic, that is, to compare the level of knowledge acquired in the absence of the father in the consultations and in their presence at prenatal surveillance consultations.

Throughout this study, it was noted that studies carried out in this area are scarce, which is why it is suggested that future investigations be carried out to assess the level of knowledge and literacy of pregnant mothers. This questionnaire proved to be a good, valid and reliable instrument for assessing a couple’s knowledge, promoting reflection by health professionals about possible improvements in their intervention.

This study can contribute to the development of future information about the level of knowledge acquired by couples through nursing care after prenatal surveillance consultations.

Given the scientific evidence presented here, it is important to evaluate, promote and improve MHL. In this way, pregnant mothers will be able to become aware of the physiological processes of pregnancy and the importance of knowing the warning signs and adhering to prenatal healthcare.

## Figures and Tables

**Table 1:** Sociodemographic characteristics.

Sociodemographic Characteristics of the Study Participants	Mother		Father	
	*N*	%	*N*	%
Marital status				
Married/cohabiting	174	88.3	174	88.3
Separated/divorced	3	1.5	2	1.0
Single	19	9.6	21	10.7
Widower	1	0.5		
Age				
21–25 years	2	1.0	2	1.0
26–30 years	39	19.8	27	13.7
31–35 years	78	39.6	65	33.0
36–40 years	65	33.0	66	33.5
41–45 years	12	6.1	26	13.2
46–50 years	1	0.5	10	5.1
Over 50 years old			1	0.5
Nationality				
Other	4	2.0	4	2.0
Portuguese	193	98.0	193	98.0
Religion				
Agnostic, Atheist, no religion	29	14.7	49	24.9
Catholic	159	80.7	139	70.6
Other	7	3.6	7	3.6
Protestant	1	0.5	1	0.5
Jehovah’s Witness	1	0.5	1	0.5
Race				
Caucasian	189	95.9	192	97.5
Black	6	3.0	3	1.5
Other	2	1.0	2	1.0
Academic Qualifications				
Basic education 2nd cycle—6th year (former 2nd year of high school/preparatory cycle)			1	0.5
Basic education 3rd cycle—9th year (former 5th year of high school or technical education)	4	2.0	7	3.6
Secondary/post-secondary education—technological specialization course	6	3.0	18	9.1
Secondary education—12th grade or equivalent	23	11.7	39	19.8
Higher education—bachelor’s degree	2	1.0	3	1.5
Higher education—doctorate	3	1.5	2	1.0
Higher education—licentiate degree	93	47.2	82	41.6
Higher education—master’s degree	66	33.5	45	22.8

**Table 2:** Obstetric and gynecologic characteristics.

Items	Mother		Father	
	*N*	%	*N*	%
Number of deliveries				
0	89	45.2		
1	83	42.1		
2	23	11.7		
3	2	1.0		
Planned pregnancy				
Yes	161	81.7	161	81.7
No	36	18.3	36	18.3
Desired pregnancy				
Yes	194	98.5	194	98.5
No	3	1.5	3	1.5
Pregnancy monitoring center				
Health center and private hospital	59	29.9		
Only in private hospital	138	70.1		
Did you take any childbirth preparation courses?				
Yes	93	47.2	72	36.5
No	104	52.8	125	63.5
Did not take the course because				
Did it during a previous pregnancy			33	16.8
COVID-19			19	9.6
Time constraints			18	9.1
We didn’t think it was necessary			19	9.6
It wasn’t in person			3	1.5
There was no availability			2	1.0
Were you accompanied by anyone during your pregnancy check-ups?				
No	192	97.5		
Yes, the husband	5	2.5		
Did you attend any pregnancy monitoring appointments?				
Yes			11	5.6
No			186	94.4
Were you present at an ultrasound scan?				
Yes			42	21.3
No			155	78.7
How many children do you have?				
1	82	41.6		
2	23	11.7		
3	6	3.0		
None	86	43.7		

**Table 3:** Mother’s knowledge.

Items	Min	Max	*A*	*SD*
The importance of vaccination	1	5	4.18	0.84
1st trimester discomfort (nausea, vomiting, constipation, drowsiness)	1	5	4.36	0.77
Need for a healthy and fractioned diet	1	5	4.31	0.78
Sexuality	1	5	3.89	1.03
Need for rest periods throughout the day	1	5	3.97	0.95
Dangers of self-medication	1	5	4.10	0.98
Body hygiene	1	5	4.12	0.99
Clothing and footwear	1	5	3.97	1.03
Layette for the maternity ward	1	5	4.07	0.96
Choosing the place of birth/booking a visit to the maternity hospital	1	5	3.93	1.01
Birth preparation courses	1	5	3.82	1.03
Advantages of breastfeeding	1	5	4.15	0.97
Preparing the nipple for breastfeeding	1	5	3.57	1.23
Correct posture during feeds	1	5	3.52	1.21
Technique, duration and intervals between feeds	1	5	3.43	1.24
2nd trimester discomforts	1	5	4.02	0.94
Signs and symptoms of urinary incontinence	1	5	3.51	1.21
Importance of preparing for birth and signs of labor	1	5	3.82	1.07
Antenatal consultation with the pediatrician	1	5	3.56	1.14
Explanation of the CTG and its importance	1	5	3.71	1.15
Teaching on how to fill in the fetal movement chart	1	5	3.62	1.10
Preparing the hospital bag	1	5	3.95	1.03
Infant deafness test	1	5	3.43	1.24
Importance of newborn monitoring	1	5	3.83	1.12
The Heel Prick Test	1	5	3.84	1.17
Vaccinating babies	1	5	3.85	1.19
Puerperium, what is it?	1	5	3.64	1.24
Lochia, puerperal depression, uterine involution, milk letdown, postpartum consultation	1	5	3.47	1.26

Note: Min = minimum; Max = maximum; *A* = average; *SD* = standard deviation.

**Table 4:** Father’s knowledge.

Items	Min	Max	*A*	*SD*
The importance of vaccination	1	5	3.78	1.17
Discomforts in the 1st trimester (nausea, vomiting, constipation, drowsiness)	1	5	3.47	1.16
Need for a healthy and fractioned diet	1	5	3.85	0.98
Sexuality	1	5	3.64	1.07
Need for rest periods throughout the day	1	5	3.69	1.06
Dangers of self-medication	1	5	3.75	1.15
Body hygiene	1	5	3.69	1.18
Clothing and footwear	1	5	3.52	1.13
Layette for the maternity ward	1	5	3.33	1.21
Choosing the place of birth/booking a visit to the maternity hospital	1	5	3.59	1.18
Birth preparation courses	1	5	3.42	1.21
Advantages of breastfeeding	1	5	3.88	1.07
Preparing the nipple for breastfeeding	1	5	2.90	1.29
Correct posture during feeds	1	5	3.01	1.29
Technique, duration and intervals between feeds	1	5	2.99	1.23
2nd trimester discomforts	1	5	3.14	1.14
Signs and symptoms of urinary incontinence	1	5	2.77	1.14
Importance of preparing for birth and signs of labor	1	5	3.35	1.21
Antenatal consultation with the pediatrician	1	5	3.16	1.31
Explanation of the CTG and its importance	1	5	3.09	1.21
Teaching on how to fill in the fetal movement chart	1	5	2.77	1.21
Preparing the hospital bag	1	5	3.28	1.30
Infant deafness test	1	5	3.14	1.31
Importance of newborn monitoring	1	5	3.70	1.15
The Heel Prick Test	1	5	3.66	1.13
Vaccinating babies	1	5	3.72	1.13
Puerperium, what is it?	1	5	2.93	1.26
Lochia, puerperal depression, uterine involution, milk letdown, postpartum consultation	1	5	2.87	1.27

Note: Min = minimum; Max = maximum; *A* = average; *SD* = standard deviation.

## Data Availability

The data presented in this study are available on request from the corresponding author.
